# Effects of *Carthamus tinctorius* L. on the ovarian histomorphology and the female reproductive hormones in mice

**Published:** 2013

**Authors:** Ali Louei Monfared, Amir Parviz Salati

**Affiliations:** 1 ***Department of Basic Sciences, Faculty of Para-Veterinary Medicine, University of Ilam, Ilam, I.R. Iran ***; 2 ***Department of Fisheries, Faculty of Marine Natural Resources, Khorramshahr University of Marine Science and Technology, Khorramshahr I.R. Iran***

**Keywords:** *Carthamus tinctorius*, Histomorphology, Mice, Ovary, Reproductive Hormones

## Abstract

**Objective:** *Carthamus tinctorius* L. (Safflower) is a member of the asteraceae family which had been classified as a fertility regulator in the traditional medicine. The purpose of this study was to investigate its possible effects on the ovarian histomorphology and the levels of female reproductive hormones in the mice.

**Materials and Methods: **Sixty adult female Balb/C mice were selected and randomly divided into one control and three experimental groups (n= 15). The control group received only distilled water, while experimental groups were administered intraperitoneally *C. tinctorius* extract at doses of 0.7, 1.4, and 2.8 mg/kg/day for 49 consecutive days. In the end of experiments, blood samples were collected and the sera were analyzed for the levels of FSH, LH, estrogen, and progesterone. Ovarian tissue samples were also taken and histomorphological changes of the ovaries were examined using optical microscope. The quantitative results were statistically analyzed by one-way ANOVA test.

**Results: **The present findings showed that treatment with different concentrations of* C. tinctorius* extract reduced the number of ovarian follicles but number of atretic follicles showed an increase. The number and size of the corpora lutea were not affected by extract administration. In addition, in the treated mice with *C. tinctorius* extract, the thickness of the tunica albuginea was increased but the relative and absolute weights of the ovaries decreased significantly. Furthermore, the blood levels of the FSH and estrogen were decreased in the three experimental groups compared with those of the control animals.

**Conclusion: **The present findings indicated that treatment with *C. tinctorius *extract has detrimental effects on the ovarian histomorphology and female reproductive hormones therefore popular consumption of this plant should be reconsidered.

## Introduction

A large number of plants have been used as therapeutic agents in traditional medicine in different countries throughout the world (Kumar et al., 2012 ▶) but there are not enough data about their probable side effects in the literature.

*C* *arthamu* *s tinctorius* (Safflower) is a member of the asteraceae family (Siddiqi et al., 2009 ▶) with traditional/folkloric use in the fertility regulation as an abortifacient agent in females for effective birth control (Kumar et al., 2012 ▶). In addition, Safflower´s flowers have applications in medicine and food industry (Elias et al., 2002 ▶; Mass, 1986 ▶). For instance, the plant is reported to have anti-inflammatory (Jun et al., 2011 ▶) and anti-tumor (Loo et al., 2004 ▶) activities and is useful in treatment of cardiomyopathy (Tien et al., 2010 ▶), gynecological disease (Zhang et al., 1998 ▶), and menstrual problems (Wang and Li, 1985 ▶) in traditional medicine.

In contrast, there are many reports indicating the toxic effects of *C. tinctorius* extract in the biological systems. For example, Louei Monfared and Salati. (2012) ▶ studied the effects of *C. tinctorius* extract administration on placental histomorphology and survival of mice neonates.

 It had been reported that *C. tinctorius *extract in doses of 1.4 and 2.8 mg/kg induces toxic changes in the placental structure and significantly decreases survival of the neonates (Nobakht et al., 2000 ▶). Association between maternal exposure to *C. tinctorius* extract and occurrence of congenital malformations in their offspring had been reported. Another study demonstrated that safflower might cause chromosomal aberrations in mouse bone marrow (Yin et al., 1991 ▶). Recently, the toxic effects of *C. tinctorius* extract on the mouse spermatogenesis and testicular tissue had been reported (Mirhoseini et al., 2012 ▶). The authors attributed the toxic effects to the action of vasodilator substances such as serotonin which present in the plant extract.

Although *C. tinctorius* is commonly used in food industry and traditional medicine, there is not enough data about the side effects of this plant on the ovarian histomorphology and the levels of female reproductive hormones. Therefore, this study was performed to investigate the eventual effects of this plant on the mouse ovary.

## Materials and Methods

*C* *arthamu* *s tinctorius* (Safflower or Golrang) plants were purchased from Emam-Reza medicinal plants market (Ilam, Iran) and botanical identification was confirmed at the herbarium of Ilam University (Herbarium number IURS-318). For extract preparation, the plant material was washed with sterile water, dried in shade at room temperature for 2 weeks, and ground in an electric mill until particles less than 4 mm were obtained. This material was extracted by maceration in 70% methanol solution at 50 ^°^C during 2 hours. The extract was filtered through a Wattman #1 paper and evaporated to dryness in a rotary evaporator under reduced pressure. 

The dried material was stored under refrigeration at 4-8 ^°^C until its use. For this study, a total of sixty adult female Balb/C mice at 29±6 grams of initial body weight and aged 12 weeks were purchased from Razi Institute (Karaj, Iran). The animals were housed in a controlled environment (temperature of 23±1 ^°^C, relative humidity 45±5%, and 12:12 h light-dark natural cycle) and had ad lib access to drinking water and food. Mice were allowed to be acclimatized to the laboratory environment at least one week before commencement of testing. Animals were randomly distributed into one control and three experimental groups, each comprising of 15 mice. The control group received only distilled water, while experimental groups were administered intraperitoneally *C. tinctorius* extract at doses of 0.7, 1.4, and 2.8 mg/kg/day for 49 consecutive days. The doses were determined on the basis of a primary study. 

In the end of the experiments, the animals were weighted and anesthetized. Then blood samples were collected via direct cardiac puncture. Serum was separated by centrifugation at 2500 rpm for 15 minutes and stored at -20 ºC until analysis. The sera were analyzed for the levels of FSH, LH, estrogen, and progesterone with radioimmunoassay method employing diagnostic kits (Immunotech, Beckman Counter Co, Czech Republic). 

For histomorphological study, the abdomen cavity was opened and the ovaries were carefully removed from the body. The obtained ovaries were trimmed out from the attached structures including fat mass and weighted using a digital scale. Then the absolute and relative weights of ovaries were determined. For optical microscopy, immersion of the ovaries was maintained overnight in neutral buffered formalin solution in order to be fixed. Then they were sectioned at 5 µm and stained with hematoxylin and eosin (H&E) and examined by Nikon microscope. Photographs was taken with a digital camera (COOLPIX 950, Nikon, China). In all groups, the number of different ovarian follicles including primordial, primary, secondary, antral, and atretic was determined using manual counting in four randomly chosen microscopic fields per ovary. In addition, the thickness of the tunica albuginea as well as the number of the corpora lutea was determined using Image Tool® 3.0 software (UTHSCSA, San Antonio, TX, USA) and compared between experimental and control groups. 

**Statistical analysis**

All results were expressed as mean ± standard error. The analysis of variance (ANOVA) was used to test the overall significance of differences among the means. Tukey-Kramer´s Multiple Comparison Test was applied for *post-hoc* comparison. Computations were performed using site-licensed SPSS statistical software (SPSS, Chicago, IL, USA). A probability level of less than 5% (p<0.05) was considered as significant. 

## Results

**Figure 1:**
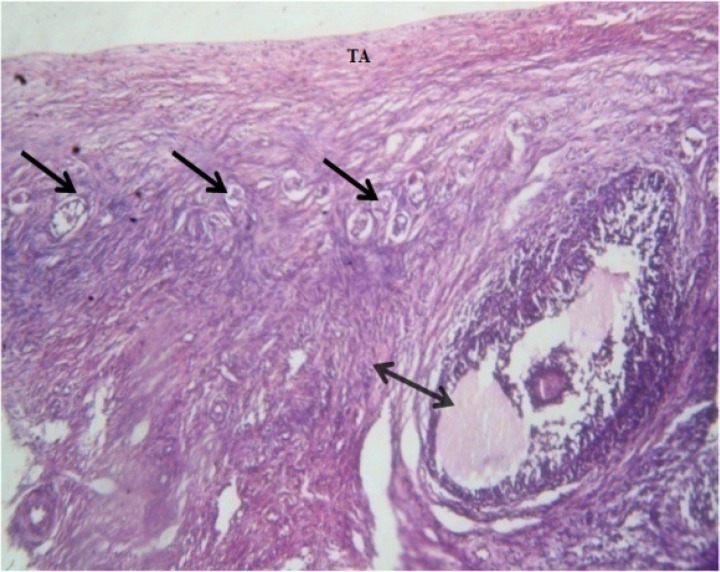
Photomicrograph of the transverse section through the ovary of the control mice that show normal structural compartments. (TA): Tunica Albuginea; (arrows): developing ovarian follicles; (double-headed arrow): Antral ovarian follicles (H&E stain: ×400).

Figure 1 illustrates the histology of the ovary of the control mice that show normal structural compartments. In these animals, the tunica albuginea had small thickness and also the population of the follicles in the cortical part of the ovary was normally high.

**Figure 2:**
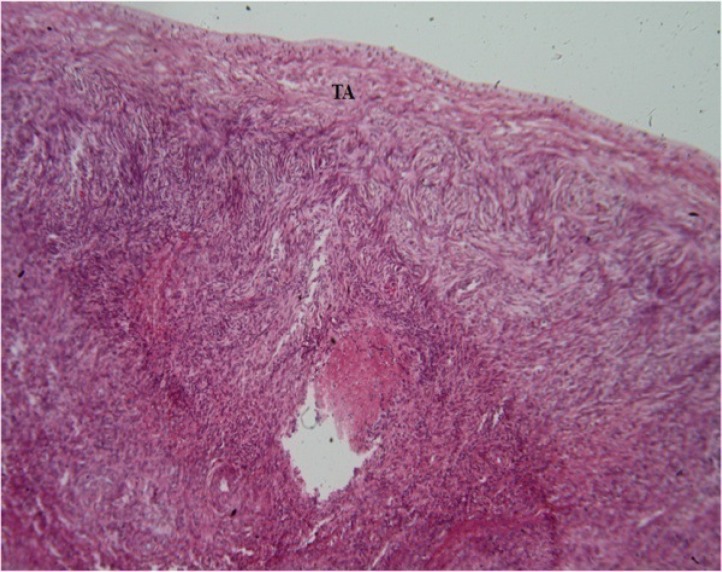
Photomicrograph of the transverse section through the ovary of the mice treated with C. tinctorius extract as 0.7 mg/kg/day. The figure shows increase in the tunica albuginea thickness and also decrease in the population of the ovarian follicles in the cortical part. (TA): Tunica Albuginea (H&E: ×400).

**Figure 3:**
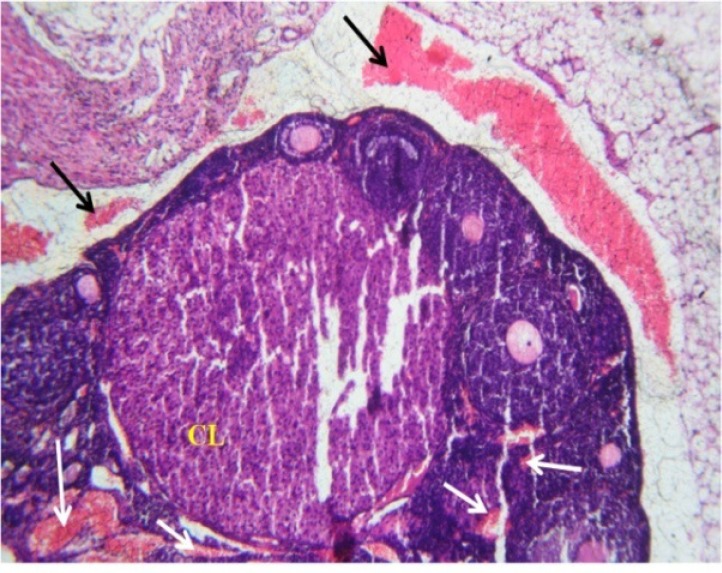
Photomicrograph of the transverse section through the ovary of the mice treated with 1.4 mg/kg/day of C. tinctorius extract. The figure shows reduction in the number of the ovarian follicles in the cortical part. Moreover, a normal corpus luteum (CL) but a multi regional congestion (arrows) is noticed in the ovarian texture. (H&E stain: ×100).

**Figure 4:**
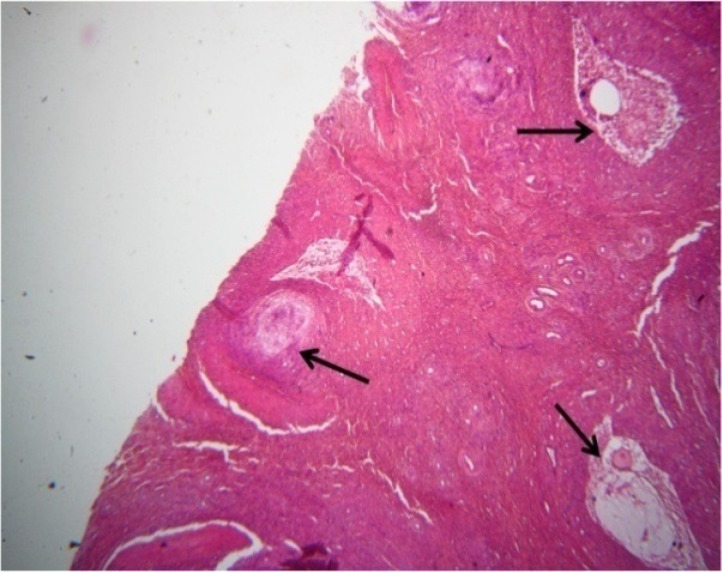
Photomicrograph of the transverse section through the ovary of the mice treated with 2.8 mg/kg/day of C. tinctorius extract. The figure shows increase of the number of the atretic follicles (arrows) in the ovarian parenchyma (H&E stain: ×400).

The effects of *C. tinctorius* extract on the histological changes of the ovary are shown in Figures 2, 3, and 4 and also Table 1. As can be seen in Table 1, treatment with different concentrations of* C. tinctorius* extract caused reduction in the number of primary, secondary, and antral ovarian follicles (p<0.05). Conversely, the number of the atretic follicles in the ovarian parenchyma was increased significantly in the treatment group (Table 1) in comparison with the control animals (p<0.05) (Figure 4). The number of primordial ovarian follicles as well as the corpora lutea count was not affected by extract administration (Table 1). In addition, in the treated mice with *C. tinctorius* extract, the thickness of the tunica albuginea was increased significantly compared with those of the controls. Furthermore, statistical analysis of the results of the ovarian weights revealed that the relative and absolute weights of the ovary in the treatment group decreased significantly in comparison with the control animals (Table 1). 

Table 2 shows the results of the female hormone profile in the control and *C. tinctorius* extract administrated animals. As can be seen in this table, treatment with *C. tinctorius* extract in the three concentrations caused a significant decrease in the serum levels of the FSH and estrogen hormones when compared with those of the control animals. The serum levels of the LH and progesterone hormones were not affected by the extract administration. No significant difference was found in the histological and female hormonal profile results between three experimental groups.

**Table 1:** Mean±SEM of the histomorphological parameters of the ovary in the control and treated mice with different doses of C. tinctorius extract (n = 15). Different letters in each rows indicate significant differences compare with control group as p<0.05

Ovarian parameters/Groups	Control	0.7 mg/kg/day of *C. tinctorius*	1.4 mg/kg/day of *C. tinctorius*	2.8 mg/kg/day of *C. tinctorius*
**Number of primordial follicles**	12.58±2.1 ^* a^	11.23±3.2 ^a^	12.12±0.33 ^a^	13.3±0.26 ^a^
**Number of primary follicles**	9.21±0.46 ^a^	5.55±1.57 ^b^	4.07±0.88^b^	5.60±2.12 ^b^
**Number of secondary follicles**	11.2±4.10 ^a^	3.6±2.3 ^b^	3.7±0.29 ^b^	3.56±0.37 ^b^
**Number of antral follicles**	9.32±2.1 ^a^	3.23±2.1 ^b^	2.5±0.11 ^b^	2.8±0.16 ^b^
**Number of atretic follicles**	1.05±0.2 ^a^	7.78±1.23 ^b^	6.8±0.27 ^b^	8.66±0.34 ^b^
**Number of corpora lutea**	2.05±0.2 ^a^	2.08±1.23 ^a^	2.1±0.27 ^a^	1.96±0.34 ^a^
**The thickness of tunica albuginea (micron)**	0.89±0.04 ^a^	3.898±1.45 ^b^	5.78±0.39 ^c^	5.37±1.29^c^
**Absolute weight of ovary (mg)**	8±0. 3 ^a^	3±0. 1 ^b^	2±0. 2 ^b^	3±0. 1^ b^
**Relative weight of ovary (ovarian weight/BW × 100)**	0.8 ^a^	0.3 ^b^	0.2 ^b^	0. 3^ b^

**Table 2:** Mean±SEM of the serum levels of female reproductive hormones in the control and treated mice with different doses of C. tinctorius extract (n = 15). Different letters used to show significant changes (p<0.05).

Female reproductive hormones /Groups	Control	0.7 mg/kg/day *C. tinctorius*	1.4 mg/kg/day *C. tinctorius*	2.8 mg/kg/day *C. tinctorius*
**FSH (U/L)**	0.4±0.09^ *^^ a^	0.09±0.01^ b^	0.05±0.08^ b^	0.06±0.01^ b^
**LH (U/L)**	0.09±0.06^a^	0.06±0.03^ a^	0.07±0.03^a^	0.09±0.07^ a^
**Estrogen (pg/ml)**	31.11±3.49^ a^	14.94±5.81^ b^	13.22±6.42^ b^	11.17±4.46^ b^
**Progesterone (ng/ml)**	35.09±4.23^ a^	34.91±3.66^ a^	33.57±4.87^ a^	32.67±6.29^ a^

## Discussion

Herbal toxicity clearly represents a serious human health threat and is an important issue to be tackled (Chen et al., 2011 ▶). On the other hand, all survey data agree that users of herbal medicine products including *C. tinctorius* are predominantly female (Eisenberg et al., 1998 ▶). It could be assumed that women frequently use herbal medicine products because they are often perceived as being “natural and therefore free of risks” (Ernst, 2002 ▶; Eisenberg et al., 1998 ▶). Therefore, it is necessary to investigate the probable side effects of the plants on the ovaries as females’ main reproductive organ and public should become aware of potential herbal toxicity.

Our findings demonstrated that intra-peritoneal injection of *C. tinctorius* in mice could induce detrimental effects on the ovarian histomorphology and female reproductive hormones. The results showed that treatment with different concentrations of* C. tinctorius* extract caused reduction in the number of primary, secondary, and antral ovarian follicles. These findings together with significant decrease in the serum levels of the FSH indicate direct toxic effects of* C. tinctorius* on the ovary structure in mice and are in line with the decrease in the serum levels of estrogen. Previously, it has been suggested that optimal blood level of FSH is known to be a prerequisite for initiation and maintenance of normal ovarian folliculogenesis (Roy and Albee, 2000 ▶). Therefore, the present histological and hormonal findings may be due to hypothalamic-pituitary-gonad axis dysfunction after treatment with the plant extract.

In this study, the number of primordial ovarian follicles was not affected by *C. tinctorius *treatment. This result may be due to the fact that formation, growth, and development of the primordial follicles in the ovary tissue initiate from perinatal period (Roy and Albee, 2000 ▶). Therefore, *C. tinctorius *treatment has not any effect on the population of these follicles. As showed in this study,* C. tinctorius *injection could induce massive follicle attrition in the ovarian parenchyma. In this regards, (Hsueh et al., 1994 ▶) showed that ovarian follicle atresia is occurred due to diminution in the blood estrogen levels, which is in line with the present hormonal results. 

In the present work, the thickness of the tunica albuginea of the ovary was increased significantly after *C. tinctorius *treatment. The normal thin tunica albuginea membrane of the ovary facilitates follicular puncture during optimal ovulation (Petyim et al., 2001 ▶) and in line with present findings it was demonstrated that in the women with anovulatory infertility, the thickness of tunica albuginea is increased due to a reduction of estradiol concentration and increase in the blood testosterone concentration (Ryzhavskiĭ et al., 2003 ▶).

The decrease in the relative and absolute weights of the ovary in the treated animals with *C. tinctorius *was the other finding in the present work. This change is resulted from the loss of follicular population in the ovary and there are similar results in the literature about ovarian weight loss due to ethanol toxicity (Van Thiel et al., 1977 ▶).

The exact mechanism of action for *C. tinctorius* ovarian toxicity in is not known according to the literatures. However, it has been reported that *C. tinctorius *plant has a variety of complex chemical constituents including flavonoids, glucosides, and rutinosides that could act on the body as a whole or on specific organs (Chen et al., 2011 ▶; Li Fan et al., 2009 ▶). Additionally, Yoo et al. (2006) ▶ demonstrated that *C. tinctorius *plant has an anti-estrogenic constituent called “tracheloside” which acts as a hormone-like agent in the Ishikawa cell system. Therefore, the above-mentioned structural changes in the ovarian structure after treatment with *C. tinctorius *extract could be a consequence of the anti-estrogenic action of this substance.Further investigations are needed to elucidate exact causative factors for *C. tinctorius*-induced ovarian toxicity. 

In conclusion, the present findings of *C. tinctorius*-induced ovarian histological changes in the mice suggest that popular consumption of this plant should be reconsidered.

## Conﬂict of interest

The authors declare that there are no conﬂicts of interest.
